# High Strain Rate Superplasticity in Al-Zn-Mg-Based Alloy: Microstructural Design, Deformation Behavior, and Modeling

**DOI:** 10.3390/ma13092098

**Published:** 2020-05-01

**Authors:** Olga Yakovtseva, Maria Sitkina, Ahmed O. Mosleh, Anastasia Mikhaylovskaya

**Affiliations:** 1 Department of Physical Metallurgy of Non-Ferrous Metals, National University of Science and Technology MISiS, Moscow 119049, Russian; yakovtseva.oa@misis.ru (O.Y.); sitkina.m@misis.ru (M.S.); 2 Mechanical Engineering Department, Shoubra Faculty of Engineering, Benha University, 108 Shoubra St., P.O., Cairo 11629, Egypt; ahmed.omar@feng.bu.edu.eg

**Keywords:** aluminum alloy, superplasticity, dynamic recrystallization, microstructural study, dislocation structure, precipitate free zone, mathematical modeling

## Abstract

Increasing the strain rate at superplastic forming is a challenging technical and economic task of aluminum forming manufacturing. New aluminum sheets exhibiting high strain rate superplasticity at strain rates above 0.01 s^−1^ are required. This study describes the microstructure and the superplasticity properties of a new high-strength Al-Zn-Mg-based alloy processed by a simple thermomechanical treatment including hot and cold rolling. The new alloy contains Ni to form Al_3_Ni coarse particles and minor additions of Zr (0.19 wt.%) and Sc (0.06 wt.%) to form nanoprecipitates of the L1_2_-Al_3_ (Sc,Zr) phase. The design of chemical and phase compositions of the alloy provides superplasticity with an elongation of 600–800% in a strain rate range of 0.01 to 0.6/s and residual cavitation less than 2%. A mean elongation-to-failure of 400% is observed at an extremely high constant strain rate of 1 s^−1^. The strain-induced evolution of the grain and dislocation structures as well as the L1_2_ precipitates at superplastic deformation is studied. The dynamic recrystallization at superplastic deformation is confirmed. The superplastic flow behavior of the proposed alloy is modeled via a mathematical Arrhenius-type constitutive model and an artificial neural network model. Both models exhibit good predictability at low and high strain rates of superplastic deformation.

## 1. Introduction

The effect of superplasticity was first described in the last century, and the nature of the phenomenon is almost clear today [1,2,3,4]. There are many works that describe this phenomenon and its feature, mechanism, and applications [5,6,7]. Owing to their fine and stable to growth grain structure and related high strain rate sensitivity of the flow stress, superplastic metallic materials demonstrate a quasi-uniform deformation with elongation-to-failure from several hundred to several thousand percent [8]. The phenomenon is used for superplastic forming (SPF), which is an effective technique of a low-volume production of complex-shape parts [9,10]. SPF allows obtaining high-quality products with very precise matrix repeatability in one processing operation using a gas in the forming process [3,10]. SPF is commercially mostly applied for aluminum- and titanium-based alloys. Several conventional superplastic aluminum alloys are manufactured, but most of them exhibit superplasticity at extremely low strain rates ≤0.002 s^−1^ [11,12]. Due to their low cost and excellent corrosion resistance, Al-Mg-based alloys are the most common ones [13,14]. Their main disadvantage is low yield strength, which is insufficient for many technical requirements [15,16]. Most of the high-strength Al-Zn-Mg-Cu-based alloys have two critical issues: (1) large residual cavitation [17,18,19] and (2) an extremely low strain rate (10^−4^ s^−1^) [20,21,22,23]. The development of aluminum alloys with high strain rate superplasticity is a challenging task [24,25,26]. The desired SPF strain rates that help to increase SPF productivity are more than 10^−2^ s^−1^ [27,28]. The strain rate above 10^−1^ s^−1^ makes the SPF method applicable for mass production [29]. 

Owing to their grain size sensitivity, the superplastic properties can be significantly improved by optimization of thermomechanical treatments [30,31] (including severe plastic deformation [32,33,34]) and chemical composition modification. Both methods focus on a grain refinement effect. The standard processing approach to obtain a fine-grained structure is cold rolling and subsequent recrystallization [2,6,12,35]. For this approach, the presence of the second phase particles of various dispersions is an important grain refinement criterion [36,37]. The coarse particles increase the nucleation rate during recrystallization due to the particle stimulated nucleation (PSN) effect [36,37,38,39,40]; fine particles inhibit a grain growth at annealing and elevated-temperature deformation due to the Zener pinning effect [37,41,42]. 

The purpose of this study is to develop a novel alloy capable of high-strain rate superplasticity and demonstrate low residual cavitation. The novel alloy provides a forming cycle reduction and expands the SPF application. This study describes the microstructure and the superplastic properties of a new high-strength Al-Zn-Mg-based alloy. The alloy is designed based on the principle of optimal microstructural heterogeneity [6,43]. An addition of nickel helps to create coarse particles of crystallization origin for PSN [44,45,46,47,48], and Sc and Zr provide the formation of nanoprecipitates for grain size stabilization via Zener pinning [48,49,50,51]. To minimize the alloy cost, the Sc content is chosen on the extremely low level of 0.06 wt.%. Notably, the sheet was processed by a simple thermomechanical treatment, including hot and cold rolling, which is applicable and inexpensive for manufacture. The optimal deformation regime including temperature, strain rate, and strain ranges should be found. The stress–strain data are required to develop pressure–time forming cycles and to simulate the superplastic forming process [10,52,53]. Mathematical models help to identify the correct SPF processing parameters and to reduce the number of experiments and the processing time [54,55]. 

The Arrhenius-type constitutive model is a phenomenological model that is generally utilized in modeling elevated temperature deformation [56,57]. This model provides very good predictability of the hot deformation behavior of various metallic materials [58,59,60,61]. The artificial neural network (ANN) exhibits an essentially different approach to simulate the flow behavior of metallic materials [62,63]. The ANN differs from statistical or numerical methods and can be used for modeling various processes without any physical principals. To minimize the variation between the inputs and the targets is the main idea of the ANN analysis. Despite the rising popularity of artificial neural networks, one should exercise caution when using them for predicting the mechanical behavior of materials. Thus, here we compare the predictability of the mechanical behavior of the studied alloy using the ANN and the classical approach based on the constitutive equations of the Arrhenius type. The development of mathematical models of superplastic deformation behavior of the studied alloy based on both Arrhenius-type constitutive hyperbolic equation (ACE) and ANN is the second important purpose of the study.

## 2. Materials and Methods

### 2.1. Preparation

The studied alloy with a chemical composition of Al–3.8%Zn–4.2%Mg–3.6%Ni–0.19%Zr–0.06%Sc (wt.%) was made from technically pure metals 99.85 wt.% Al, 99.92 wt.% Zn, and 99.95 wt.% Mg and master alloys of Al-18 wt.%Ni, Al-3.5wt.%Zr, and Al-2wt.%Sc. The alloy was melted in a Nabertherm S3 laboratory electric furnace in a graphite–fireclay crucible. The casting temperature was about 770 °C. The ingot obtained by casting in a water-cooled copper mold with internal dimensions of 100 × 40 × 20 mm^3^ provided a solidification rate of ~15 K/s. Homogenization annealing was performed in three steps at temperatures of 360 ± 2 °C for 6 h, 450 ± 2 °C for 3 h, and 500 ± 2 °C for 2 h in a Nabertherm N30/65A furnace with air convection. The first annealing step aimed to precipitate the coherent Al_3_(Sc,Zr) nanoscale dispersoids, and the second one aimed to dissolve the nonequilibrium T(Al_2_Mg_3_Zn_3_) phase. The third step provided the spheroidization and fragmentation of the Al_3_Ni particles as well as the elimination of dendrite liquation of the aluminum solid solution. Hot rolling performed at 420 ± 10 °C to a thickness of 3 mm followed rolling at room temperature to process a sheet thickness of 1 mm. The incipient melting/solidus temperature (T_i.m._) of the alloy was 552 °C; it was determined by the differential thermal analysis using a Setaram Labsys DSC 1600 calorimeter with a cooling rate of 5 K/s. 

### 2.2. Microstructural Analyses

The microstructure and the local chemical composition were controlled using a TESCAN Vega 3 LMH scanning electron microscope (SEM, Tescan Brno s.r.o., Kohoutovice, Czech Republic) with an X-Max 80 energy-dispersive detector. The SEM imaging was conducted using secondary electron and backscattered electron scanning regimes. An Axiovert 200 MMat light/optical microscope (LM, Carl Zeiss Oberkochen, Germany) was used to analyze the grain structure in polarized light. The specimens were cut from the upper and lower parts of the ingot and from several parts of the rolled sheet. The specimens were prepared by mechanical grinding by a SiC paper and the final polishing with a colloidal silica suspension using a Struers LaboPol machine (Struers ApS, Ballerup, Denmark). The grain structure was analyzed after an anodic oxidation in a 10% fluoroboric acid water solution. The volume fractions, the size, the shape of the particles, and the cavity volume fraction were determined via analyses of 7–8 micrographs using an AxioVision 4.5 software (Carl Zeiss Oberkochen, Germany).

The dispersoid parameters and the dislocation structure were examined using a JEOL JEM-2000EX transmission electron microscope (TEM, Tokyo, Japan). The specimens for TEM were discs of 3 mm in diameter mechanically pre-ground to a thickness of 0.22 mm. The finish step of the TEM-specimens preparation was electropolishing in a Struers TenuPol-5 (Struers ApS, Ballerup, Denmark) device using a 70% CH_3_OH + 30% HNO_3_ electrolyte at a temperature of 253 K and a polishing voltage of 19.5 V. 

### 2.3. Superplastic Parameters

The specimens for the elevated temperature tensile tests were cut along the rolling direction of the sheet; the gauge size had a length of 14 mm and a width of 6 mm. The superplastic parameters were determined via a step-by-step change in the strain rate tests and constant strain rate tests. The strain rate was maintained constant because the traverse speed increases proportionally as the specimen length increases. The tests were performed in an argon atmosphere on a Walter Bay LFM-100 testing machine with Dion-Pro software (Walter + Bai AG, Löhningen, Switzerland), which allowed programing the traverse motion during the experiment. The step-by-step increase strain rate tests were done in a temperature range of 400 to 520 °C after pre-straining to e = 0.69 (100%) at a low strain rate of 0.002 s^−1^; this strain is required to the formation of a near-recrystallized grain structure. The m-index vs. strain rate curves were achieved by differentiation of the stress–strain rate logarithmic curves. The constant strain rate tests were performed in a strain rate range of 0.002 to 1 s^−1^ and a temperature range of 400 to 520 °C. 

### 2.4. Modeling

The experimental data obtained by the constant strain rate tests at different deformation temperatures and strain rates were used to construct the Arrhenius-type constitutive hyperbolic equation (ACE) and the artificial neural network (ANN) models [64]. The sequences of building each model were described in detail in the previous works [65,66]. The performance of the constructed models was assessed by calculating the correlation coefficient (R) (Equation (1)), the root mean square error (RMSE) (Equation (2)), and the average absolute relative error (AARE) (Equation (3)) [67,68].

(1) $$R=\frac{\sum_{i=1}^{N}\left(E_{i}-\bar{E}\right)\left(P_{i}-\bar{P}\right)}{\sqrt{\sum_{i=1}^{N}{\left(E_{i}-\bar{E}\right)}^{2}\sum_{i=1}^{N}{\left(P_{i}-\bar{P}\right)}^{2}}}\;\;$$

(2) $$RMSE=\sqrt{\frac{1}{N}\overset{N}{\underset{i=1}{\sum }}{\left(E_{i}-P_{i}\right)}^{2}}\;$$

(3) $$AARE=\frac{1}{N}\overset{N}{\underset{i=1}{\sum }}\left|\frac{E_{i}-P_{i}}{E_{i}}\right|\;\;\;\;\;$$

where 

*E_i_,* and *P_i_* are the experimental and modeled flow stress, respectively;

$\bar{E}$ and $\bar{P}$ are the mean values of the experimental and modeled flow stress, respectively; and

*N* is the sample size.

The predictability of the constructed ACE and ANN models was evaluated by comparing the experimental results obtained at a temperature of 440 °C and a strain rate of 0.01 s^−1^ and 0.1 s^−1^ (these conditions were not included within the input conditions for constructing both models) with the predicted values from both models.

## 3. Results and Discussion

### 3.1. Subsection

**Figure 1:**
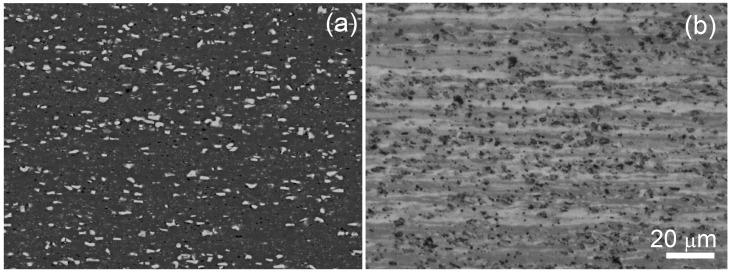
Initial microstructure after (**a**) cold rolling, SEM; (**b**) LM-image after annealing at a temperature of 520 °C for 30 min.

The mean size of Al3Ni eutectic particles was 0.82 ± 0.15 µm, the form factor of 0.92 and their fraction was 9 ± 2% (Figure 1a). After annealing in a temperature range of 400 to 520 °C (0.86–0.96T_i.m_.) for 30 min, the grain structure consisted of the banded grains of a mean size 2.8 ± 0.2 μm (Figure 1b). 

**Figure 2:**
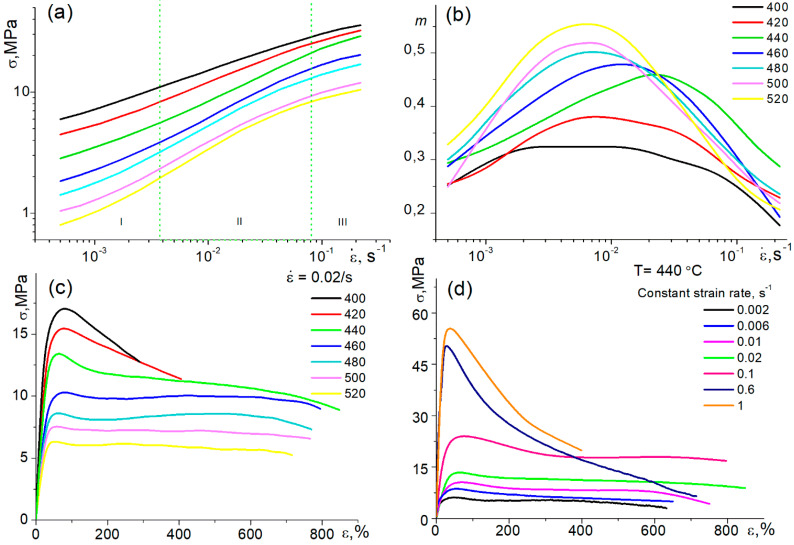
(**a**) Stress–strain rate logarithmic curves and (**b**) strain rate sensitivity m-index-strain rate semilogarithmic curves in a temperature range of 400 to 520 °C (the samples were pre-deformed to 0.69 at a constant strain rate of 0.002 s^−1^ at each temperature); (**c**) stress–strain curves at a range of 400 to 520 °C and a constant strain rate 0.02 s^−1^; (**d**) stress-strain curves at a temperature of 440 °C and different strain rates.

Figure 2a shows the stress–strain rate logarithmic curves that have a typical superplastic behavior sigmoidal shape that consists of three regions [2,6,8,69]. The second liner region corresponds to superplastic behavior [2,8]. In a temperature range of 440 to 520 °C, the maximum strain rate sensitivity m-index ranged from 0.45 to 0.55 (Figure 2b). With decreasing the deformation temperature from 520 to 440 °C, the maximum m decreased, while its strain rate position indicating an optimum strain rate value increased. The maximum optimum strain rate of 0.02 s^−1^ (maximum-m position) and the maximum elongation-to-failure of 854 ± 21% during the constant strain rate tests (Figure 2c,d) were achieved at 440 °C. The alloy exhibited a stable flow with an elongation of 700–800% at a constant strain rate of 0.02 s^−1^ in a temperature range of 440 to 520 °C (Figure 2c), and the same elongation values at 440 °C in a constant strain rate range of 0.01 to 0.6 s^−1^ (Figure 2d). It is notable that the evidence of superplasticity with a mean elongation-to-failure of 402 ± 28% was detected at an extremely high constant strain rate of 1 s^−1^. The near-superplastic behavior was observed at lower temperatures of 400–420 °C, but the maximum m-value reached only 0.32–0.37, and a mean elongation-to-failure at a strain rate of 0.02 s^−1^ was ≤400%.

**Figure 3:**
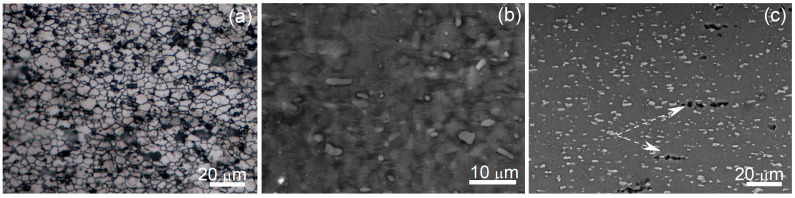
Microstructures after superplastic deformation: (**a**) LM image of the grain structure after failure at 420 °C and 0.02 s^−1^; (**b**) SEM image of the deformed at 0.02 s^−1^ and 440 °C to strain of 0.69 pre-polished surface; (**c**) SEM microstructure after failure at e = 2.2 at a strain rate of 0.02 s^−1^ at 440 °C; the white arrows indicate cavities; the tensile direction is horizontal.

Most of the stress–strain curves in Figure 2c,d can be divided into two main parts: (1) the peak stress at low strains of ~30–50% with strain softening up to 100% (e = 0.69) and (2) the near-stable flow accompanied insignificant strain softening/hardening. At a temperature of 440 °C and a high strain rate of 0.1 s^−1^, softening occurred at a larger strain of 250%. At a low temperature of 400–440 °C and strain rates of 0.6 and 1 s^−1^, the deformation was accompanied by softening up to-failure (see the red and black curves in Figure 2c and the dark-blue and orange curves in Figure 2d). As the deformation was quasi-uniform, the strain rate was constant at testing, and the initial grain structure was unrecrystallized, such stress–strain behavior can be related to a dynamic recrystallization [17,37,70]. At lower temperatures and higher strain rates, the dynamic recrystallization needs larger strains and more time to complete, and prolonged strain softening was observed. The microstructural studies confirmed the transformation of the initial banded grains into equiaxed dynamically recrystallized ones (see the example of the microstructure of the samples deformed to failure at 420 °C and 0.02 s^−1^ in Figure 3a).

**Figure 4:**
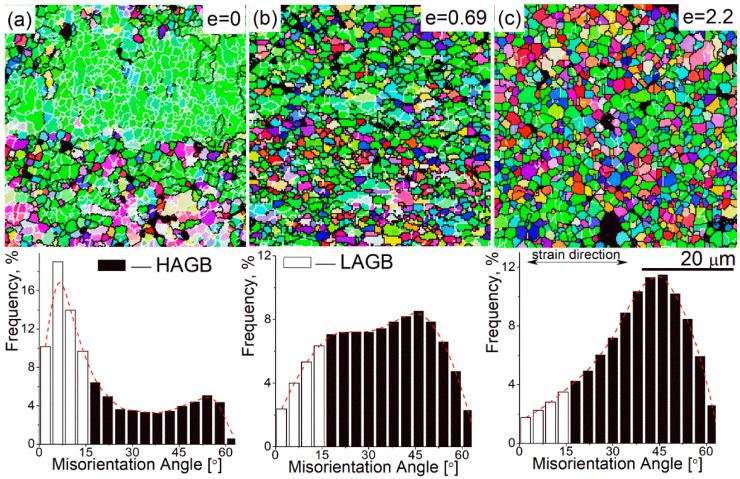
SEM-electron backscatter diffraction (EBSD) grain boundaries maps (the low-angle grain boundaries (LAGBs) are white colored and the high-angle grain boundaries (HAGBs) are black colored in the maps), and the angle misorientation of the samples deformed to 0 (**a**), 0.69 (**b**), 2.2 (**c**) at 0.02 s^−1^ and 440 °C.

The strain-induced microstructural evolution was carefully studied at a temperature of 440 °C and a strain rate of 0.02 s^−1^; those deformation conditions corresponded to the maximum strain rate sensitivity and the maximum elongation-to-failure. A typical superplastic deformation surface relief related to grain boundary sliding was revealed on the pre-polished sample deformed to 0.69 (Figure 3b). A high fraction of 60% of low-angle grain boundaries (LAGB, 2–15°) and a small fraction of 40% of high-angle grain boundaries (HAGB ≥15°) were observed before the start of superplastic deformation (Figure 4a). The LAGB fraction significantly decreased and the HAGB proportionally increased to 82% after 0.69 of strain (Figure 4b), and to 90% after 2.2 of strain (Figure 4c). Thus, the strain softening at the beginning of the deformation is explained by the dynamic recrystallization process, which was stimulated by PSN in the presence of Al_3_Ni particles [45,46]. There was no significant grain growth at deformation; the mean grain size was 1.9 ± 0.4 μm after a strain of 0.69 and 2.4 ± 0.3 μm after failure at a strain of 2.2 (Figure 4). Therefore, a fine-grained structure is formed at superplastic deformation and the grains demonstrate increased size stability to dynamic growth.

**Figure 5:**
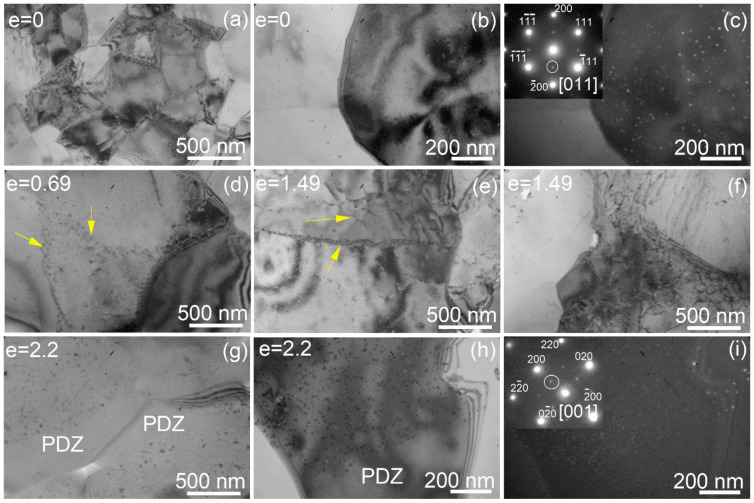
TEM structures after 20 min of annealing at 440 °C (**a**–**c**), after strain 0.69 (**d**), 1.49 (**e**,**f**), and 2.2 (**g**–**i**) at a temperature of 440 °C and a strain rate of 0.02 s^−1^; the yellow arrows show the dislocation walls.

The dislocation structure was studied before the start of superplastic deformation and after the deformation with strains of 0.69, 1.49, and 2.2 (Figure 5). The initial microstructure consisted of subgrains and grains with a low amount of dislocations inside them (Figure 5 a–c). Many dislocation walls were observed after 0.69 and 1.49 of strain (Figure 5d–f, arrows). After a strain of 2.2 near failure, high-angle grain boundaries were observed, and the grains were almost free of dislocations. The precipitation-depleted zones (PDZ) were identified on both sides of the HAGB boundaries and in triple junctions (Figure 5g–i). This phenomenon typically associates with the diffusion creep mechanism that can accommodate the grain boundary sliding and be an important independent deformation mechanism [71]. 

High dense distributed L1_2_-structured nanoprecipitates of the Al_3_(Sc,Zr)-phase were observed before the start of the deformation and after failure. Their structural type was unchanged at superplastic deformation, whereas their mean size insignificantly grew from 17 ± 4 nm before the start of the deformation to 22 ± 5 nm after failure at e = 2.2 (Figure 5). Thus, the stable grain structure at superplastic deformation is a result of high density of thermally stable nanoprecipitates of the L1_2_-Al_3_(Sc,Zr) phase providing the Zener drag.

Superplastic deformation was accompanied by cavitation, which is typical of aluminum-based alloys [18,21,72]. As the grain boundary sliding (GBS) is a dominant superplastic deformation mechanism [71,72,73], the coarse secondary particles on the grain boundaries are expected to act as nucleation sites for strain-induced cavities [72]. In the studied alloy, the cavities formed near the eutectic particles and far from them (Figure 3c), but the volume fraction of the residual cavities reached only 1.2% after deformation to failure. This value of residual cavitation is several times lower compared with that of in the AA7475 alloy [71]. Despite the 10% of Al_3_Ni coarse particles, the residual cavitation was insignificant in the designed alloy. First, a cause of low residual cavitation is a compact near-spherical shape of the Al_3_Ni particles. Second, the local lattice misorientations with increased dislocation density, which formed near coarse and hard Al_3_Ni particles, provide a formation of new grains surrounded by HAGB during the superplastic flow [11,45]. It is suggested that low cavitation can be a result of a rapid accommodation of the GBS in a fine-grained structure, including the places near the coarse particles. As it was shown in [46,74], the superplastic properties of the Al-Zn-Mg-Cu-Zr-Sc alloy were also significantly improved due to the presence of the coarse Ni-bearing particles.

Due to its microstructural design, in the presence of the coarse Al_3_Ni particles of eutectic origin and the L1_2_-Al_3_(Sc,Zr) nanoprecipitates, the developed alloy exhibits a stable fine-grained structure at superplastic deformation, low residual cavitation, and high strain rate superplasticity up to a strain rate of 1 s^−1^.

### 3.2. Flow Stress Behavior Modeling

#### 3.2.1. Arrhenius-Type constitutive hyperbolic Equation model (ACE)

The flow stress (σ) depends on the deformation temperature (T), strain rate ($\dot{\varepsilon }$), and strain (ε) as follows (Equation (4)) [75],

(4) $$\sigma =f\left(T,\dot{\varepsilon },\;\varepsilon \right).$$

The Zener–Holloman parameter (Z) in the exponent-type formula is used to describe the relationship between T, $\dot{\varepsilon }$, and ε (Equation (5) and (6)) [58,75,76],

(5) $$Z=\dot{\varepsilon (}exp^{\left(\frac{Q}{RT}\right)})$$

(6) $$\begin{array}{cc} \dot{\varepsilon }=Af\left(\sigma \right)\left({exp}^{\left(-\frac{Q}{RT}\right)}\right)=\;\varepsilon^{.} & \\ & =\{\begin{array}{l} A_{1}\sigma^{n_{1}}(exp^{\left(-\frac{Q1}{RT}\right)})-\text{P}ower\;law\\ A_{2}(exp^{\left(\beta \sigma \right)})(exp^{\left(-\frac{Q2}{RT}\right)})-Exponential\;law\;\\ A_{3}{\left[\sinh\left(\alpha \sigma \right)\right]}^{n_{2}}\left(exp^{\left(-\frac{Q3}{RT}\right)}\right)-Hyperbolic\;sine\;law \end{array} \end{array}$$

where

*A_1_*_,*2*,*3*_, *α (*α=*β/*$n_{1})$, *β*, $n_{1}$, *n_2_* are the material constants that depend on the effective strain

*Q_1_*_,*2*,*3*_ are the thermal deformation activation energy in J/mol

*T* is the absolute temperature in K

*R* is the universal gas constant, 8.314 J/(mol·K).

Due to their high efficiency in wide ranges of stress levels, the hyperbolic sine law is utilized to construct the proposed constitutive model in this work [56,58,63]. 

#### 3.2.2. Determination of the Constitutive Model Constants

The procedure of determining the constitutive model constants (α, Q_3_, ln(A_3_), and n_2_) is illustrated in detail in [65,66]. On the bases of the hyperbolic sin law, the relationship between $\dot{\varepsilon }$, T, and σ can be expressed as follows (Equation (7)),

(7) $$\dot{\varepsilon }=A_{3}{\left[\sin h\left(\alpha \sigma \right)\right]}^{n_{2}}exp^{\left(-\frac{Q3}{RT}\right)}$$

The flow stress can be written as a function in the Zener–Holloman parameter according to the hyperbolic law (Equation (8)).

(8) $$\sigma =\frac{1}{\alpha }\ln\left\{{\left(\frac{\dot{\varepsilon }exp^{\left(\frac{Q3}{RT}\right)}}{A_{3}}\right)}^{\frac{1}{n_{2}}}+{\left[{\left(\frac{\dot{\varepsilon }exp^{\left(\frac{Q3}{RT}\right)}}{A_{3}}\right)}^{\frac{2}{n_{2}}}+1\right]}^{\frac{1}{2}}\right\}$$

**Figure 6:**
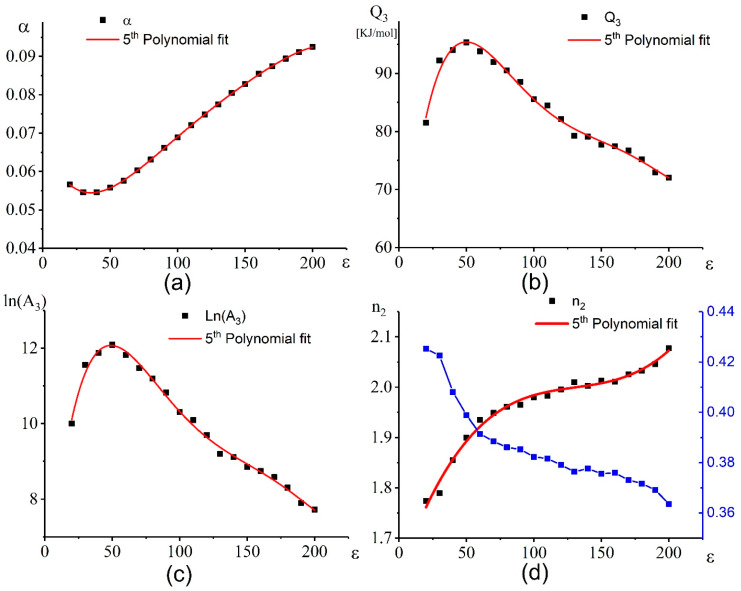
Dependence of the (**a**) α, (**b**) Q_3_, (**c**) ln(A_3_), and (**d**) n_2_ on strain.

Figure 6 shows the dependences of the α (Figure 6a), *Q_3_* (Figure 6b), ln(A_3_) (Figure 6c), and n_2_ (Figure 6d) on strain. 

Based on the correlation coefficient (R), the most appropriate polynomial order is the 5th order. The coefficients of the equations for the all parameters are illustrated as follows (Equations (9)–(12)) [65,76].

(9) $$\alpha =a+b\;\left(\varepsilon \right)+c\;\left(\varepsilon^{2}\right)+d\;\left(\varepsilon^{3}\right)+e\;\left(\varepsilon^{4}\right)+f\;\left(\varepsilon^{5}\right)$$

(10) $$Q_{3}=a+b\;\left(\varepsilon \right)+c\;\left(\varepsilon^{2}\right)+d\;\left(\varepsilon^{3}\right)+e\;\left(\varepsilon^{4}\right)+f\;\left(\varepsilon^{5}\right)$$

(11) $$\mathrm{Ln}\left(A_{3}\right)=a+b\;\left(\varepsilon \right)+c\;\left(\varepsilon^{2}\right)+d\;\left(\varepsilon^{3}\right)+e\;\left(\varepsilon^{4}\right)+f\;\left(\varepsilon^{5}\right)$$

(12) $$n_{2}=a+b\;\left(\varepsilon \right)+c\;\left(\varepsilon^{2}\right)+d\;\left(\varepsilon^{3}\right)+e\;\left(\varepsilon^{4}\right)+f\;\left(\varepsilon^{5}\right)\;$$

The coefficients (a, b, c, d, e, and f) and the R^2^ of the 5th-order polynomial equation for each material constant are given in Table 1.

**Figure 7:**
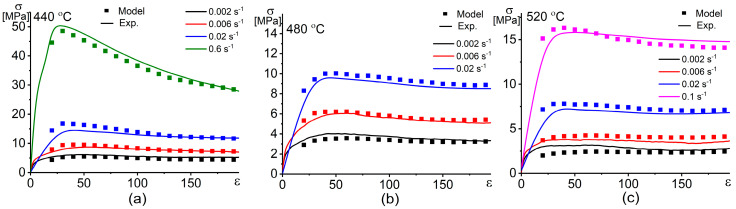
Dependences of the experimental and the predicted by the Arrhenius-type constitutive hyperbolic equation (ACE) model stresses vs. strain at different strain rates and temperatures of (**a**) 440 °C, (**b**) 480 °C, and (**c**) 520 °C.

Once the material constants are evaluated via Equations (9)–(12), the flow stress at an effective strain rate is predicted using Equation (8). Figure 7 shows the stress–strain dependence at different temperatures and strain rates of the investigated alloy.

#### 3.2.3. Artificial Neural Network Model (ANN)

**Figure 8:**
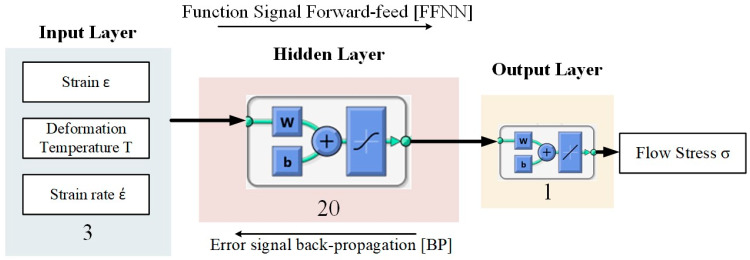
Schematic illustration of the artificial neural network (ANN) architecture of the used network.

The ANN is widely used for modeling and simulating the flow behavior of metallic materials due to its simplicity and fast response. Thus, the main aim here is to compare the predictability of the mechanical behavior of the studied alloy using the ANN and the classical approach based on the constitutive equations of the Arrhenius type. In this work, a three-layer network with the backpropagation algorithm was used to model the flow stress of the investigated alloy. The deformation temperature, the deformation strain rate, and strain are chosen as the inputs, and the flow stress is set as the output in the model. The ANN architecture is schematically illustrated in Figure 8. The setting of other training parameters for the neural network is listed in Table 2 according to [65,66].

**Figure 9:**
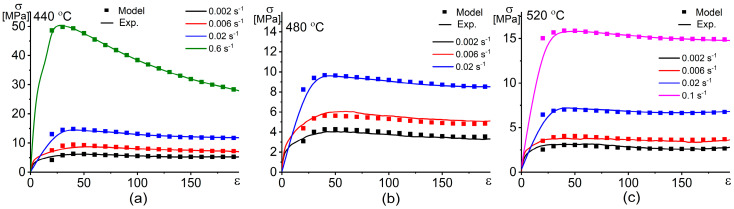
Dependences of the experimental and the predicted in the ANN model stresses vs. strain at different strain rates and temperatures of (**a**) 440 °C, (**b**) 480 °C, and (**c**) 520 °C.

**Figure 10:**
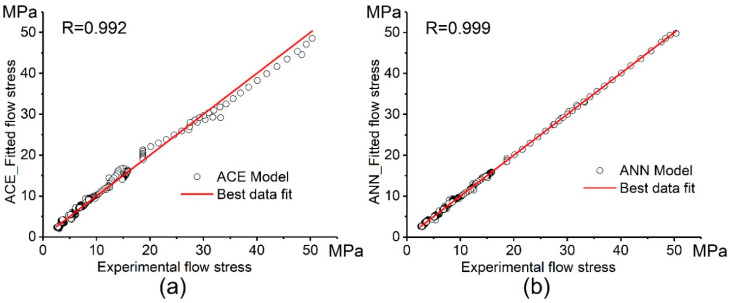
Correlation between the experimental and the modeled flow stress obtained by the (**a**) Constitutive model and (**b**) ANN model.

Figure 9 shows the correlation between the experimental flow stress and the modeled flow stress obtained by the constitutive model and the ANN model at different strain rates and temperatures. The correlation between the experimental flow stress and the modeled flow stress via both ACE and ANN models is shown in Figure 10. After fitting and approximation, the values of the performance indicators R, AARE (%), and RMSE for the ACE model are 0.992, 6.0%, and 0.95, respectively. These values for the ANN model are 0.999, 2.5%, and 0.45, respectively. Both the Arrhenius-type constitutive model (ACE) and the artificial neural network (ANN) exhibit good workability in modeling and predicting the flow stress of the investigated alloy; however, the ANN is more accurate than the ACE.

#### 3.2.4. Models Verification

**Figure 11:**
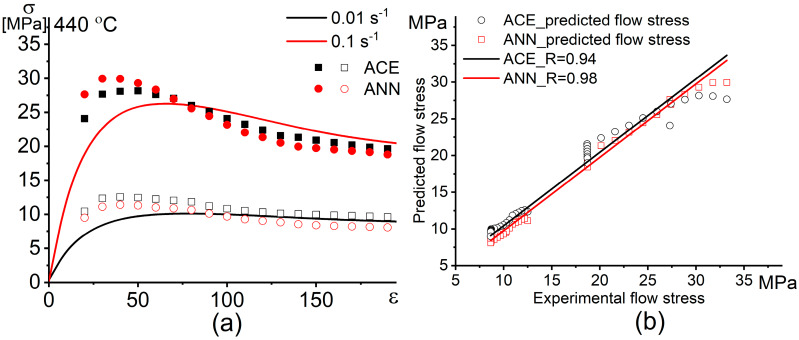
(**a**) Dependence and (**b**) correlation of the experimental and the predicted flow stress obtained by the constitutive model (CE) and the ANN model vs. strain.

To evaluate the quality and the performance of the constructed models, more experimental results obtained from the isothermal tensile tests at 440 °C, 0.01 s^−1^, and 0.1 s^−1^ were used to test both models (these conditions are not included in the input conditions for constructing both models). Figure 11 shows the comparison and correlation between the experimental flow stress and the predicted flow stress for both models. After the prediction of unmodeled conditions, the values of the performance indicators R, AARE (%), and RMSE for the ACE model are 0.94, 1.4%, and 0.5, respectively, whereas these values are 0.98, 1.1%, and 0.4 for the ANN model, respectively. The results revealed excellent efficiency of the constructed models for high and low strain rates.

Figure 11 demonstrates a minor difference between the predictability of the flow stress using a classical approach based on the Arrhenius type constitutive equation and the Artificial neural network. Thus, the ANN can be used for modeling of the superplastic flow behavior as a simple modeling approach. 

## 4. Conclusions

The superplastic deformation behavior and strain-induced microstructural evolution of the novel Al-3.8%Zn-4.2%Mg-3.6%Ni-0.19%Zr-0.06wt.%Sc alloy were studied by optical, scanning, and transmission electron microscopy techniques. Sheets were processed by a simple thermomechanical treatment. The mathematical models of the stress–strain behavior were developed. The following conclusions are summarized.

1. Before the start of superplastic deformation in a temperature range of 400 to 520 °C (0.86–0.96T_i.m._), the microstructure of the cross-thickness sheet section consists of un-recrystallized banded grains of 2.8 µm thickness, ~10% of the Al_3_Ni coarse particles of eutectic origin of 0.8 µm mean size, and nanoprecipitates of L1_2_-Al_3_(Sc,Zr) phase with a mean size of ~20 nm. 

2. The alloy demonstrates superplasticity in a strain rate range from traditionally low 0.002 s^−1^ to an extremely high strain rate of 1 s^-1^ in a temperature range of 400 to 520 °C with elongation-to-failure of 400–800% and strain rate sensitivity index m > 0.3. 

3. The analyses of the strain-induced microstructural evolution at an optimal superplastic temperature of 440 °C and a strain rate of 0.02 s^−1^ revealed a formation of dislocation walls, an increasing of the high angle grain boundaries fraction at low strains, low residual cavitation <2%, weak dynamic grain growth, and a stable size and structure of the L1_2_-Al_3_(Sc,Zr) nanoprecipitates. The formation of precipitate-depleted zones on the grain boundaries and triple junctions, which suggested a diffusional creep deformation mechanism, was revealed. 

4. Due to its microstructural design, in the presence of the coarse Al_3_Ni particles provided PSN, and the L1_2_-Al_3_(Sc,Zr) nanoprecipitates provided grain size stabilization via the Zener pinning effect, the designed alloy exhibits a stable fine-grained structure, low residual cavitation at superplastic flow and, as a result, high strain rate superplasticity.

5. After both fitting and the prediction of unmodeled conditions, the mathematical Arrhenius-type model and the artificial neural network model exhibited good workability. The average absolute relative error between the experimental and the predicted flow stress did not exceed 1.5% for both models. Therefore, both models efficiently approximate and predict the flow behavior of the alloy under superplastic deformation at high and low strain rates.

6. Extraordinarily high strain rate superplasticity of the alloy processed by a simple thermomechanical treatment as well as low scandium content in this alloy makes the novel alloy an attractive material for superplastic forming. Further experiments should be focused on determining the dominant deformation mechanism of high strain rate superplasticity. A role of each microstructural parameter in the deformation behavior needs to be identified. It helps to develop new aluminum-based alloys with high strain rate superplasticity.

## Figures and Tables

**Table 1:** The calculated coefficients in Equations (9)–(12).

Coefficients	α	$Q_{3}$	$Ln\left(A_{3}\right)$	$n_{2}$
**a**	0.06794	47.62414	4.73584	1.62913
**b**	−8.99155 × 10^−4^	2.54795	0.39416	0.00757
**c**	1.92787 × 10^−5^	−0.0476	−0.00737	−5.09154 × 10^−5^
**d**	−1.49955 × 10^−7^	3.81136 × 10^−4^	5.83719 × 10^−5^	8.17962 × 10^−8^
**e**	5.68411 × 10^−10^	−1.42221 × 10^−6^	−2.15681 × 10^−7^	3.02081 × 10^−10^
**f**	−8.64638 × 10^−13^	2.01632 × 10^−9^	3.02832 × 10^−10^	−5.38008 × 10^−13^
**R^2^**	0.999	0.991	0.995	0.983

**Table 2:** Settings of the training parameters for the used neural network.

Parameters	Contents
Network	Backpropagation Network (BP)
Function	TrainLM
Transfer function of output layer	Liner (purelin)
Transfer function of hidden layer	Tan-sigmoid
Training epoch	8000
Goal	1 × 10^−6^
Performance function	MSE
